# Supplementary material for: Are carbon emissions trading and green financial instruments synergistic? -Comprehensive quantitative research based on content analysis
Source: PLoS One. 2024 Mar 7;19(3):e0298601. doi: 10.1371/journal.pone.0298601 (PMC10919601; doi:10.1371/journal.pone.0298601)
Supplement: S1 Data — (ZIP) [file pone.0298601.s001.zip › Data/地级市Z_F工作报告.zip!/║╙─╧/║╫▒┌╩╨/└┤╫╘╣1⁄2╓┌║┼í╛┬φ┐╦╩2╛▌═°í┐_2010.docx]

各位代表：

　　现在，我代表市人民政府，向大会作工作报告，请予审议，并请各位政协委员提出意见。

　　一、2009年工作回顾

　　2009年是新世纪以来我市经济社会发展最为困难的一年，也是全市人民团结一心，战胜严峻考验、取得重大成效的一年。在省委、省政府和市委的正确领导下，全市上下深入贯彻落实科学发展观，迅速全面进入"战时状态"，采取一系列超常措施，积极应对国际金融危机带来的前所未有的压力和挑战，最大限度减轻了危机对我市的不利影响，较好地完成了市九届人大一次会议确定的各项任务，取得了保增长、保民生、保稳定的重大胜利，在加快两个构建、实现率先崛起进程中迈出了坚实步伐。经济平稳较快增长，企稳回升态势好于预期。全年生产总值377.5亿元，比上年增长12.8%；财政一般预算收入18亿元、增长13.4%，支出46.3亿元、增长42.9%。跨越发展保持良好态势，谋划实施了一大批促进跨越发展的重大项目，开展了大量事关全局的基础性工作，新型产业基础加快构建，新型城市骨架雏形初显。民生进一步改善，在财政极其困难的情况下，投入资金19.6亿元、占财政支出的42.3%，圆满完成了省"十大实事"涉及我市的任务和我市12项民生工程年度建设任务。预计农民人均纯收入、城镇居民人均可支配收入增长8%和11%。社会大局和谐稳定，坚持不懈抓好安全生产和社会稳定，在各类矛盾明显增多的形势下，实现了社会治安形势、信访形势和安全生产形势基本平稳，被评为全国社会治安综合治理优秀市、全省信访工作先进市。

　　一年来，主要抓了以下几个方面的工作：

　　(一)下大力气推进项目建设，良好发展态势进一步巩固和强化。把项目建设作为各项工作主抓手，扎实开展"项目建设年"活动，围绕全市煤电化材一体化、食品加工、机械制造、金属镁精深加工、电子信息等战略支撑产业和新兴先导产业，组织实施了一批对全市发展具有重要支撑和带动作用的项目，项目建设取得历史性突破。全市确定重点项目160个，完成投资150亿元，是上年的2倍多；新开工项目106个，是上年的近4倍；竣工项目36个，是上年的2倍多。6个省重点项目投资进度居全省第2位；108个列入省"8511"投资促进计划、"1074"计划和"双百计划"项目，投资进度均居全省前列；中央新增投资项目进展顺利。全力服务南水北调中线工程、石武客运专线等过境项目建设，保障了工程快速推进与社会和谐稳定。重点项目建设的强力推进，有效地拉动了全市经济社会发展，城镇固定资产投资全年增长34.4%；战略支撑产业和新兴先导产业对工业投资、规模以上工业增加值、工业税收的贡献率均超过90%，支撑作用进一步增强。在立足当前保增长的同时，着眼长远打基础，设立产业发展基金，集中扶持重点产业、新技术新产品研发和结构调整等；投入较大精力谋划制定并大力实施煤电化材等多个产业发展规划，进一步理清了发展思路，明确了发展方向和目标，增强了对经济社会发展的引领性。

　　(二)把招商引资放在事关全局的高度来部署，经济社会发展后劲明显增强。大力加强与央企的战略合作，积极承接沿海地区产业转移，突出抓好行业龙头和产业集群的引进，着力提升产业配套能力，成功举办了第十二届中国镁协年会暨首届镁应用新产品新技术展示会、中国(鹤壁)电子信息产业高峰论坛等一系列规模和影响较大的招商活动，一批产业支撑项目相继落地，实际利用外商直接投资和省外资金均提前超额完成省定目标任务。与中石化合作的大型煤制烯烃等重大项目正在积极推进；石林陶瓷产业集聚区已签约总投资超过40亿元的46条陶瓷生产线，入驻11家陶瓷及配套企业。积极争取上级资金19.4亿元，争取国债资金总额居全省前列。

　　(三)及时深入开展企业服务年活动，工业形势整体好转。面对年初全市近50%的企业停产、半停产的严峻形势，在全省率先开展企业帮扶工作，及时出台了23个帮扶企业渡难关、支持企业做大做强的政策性文件，认真排查、及时解决企业反映突出的资金、土地、用工等371个问题，协调周转资金60多亿元，及时落实优惠政策，减免税费2.2亿元。鼓励引导金融机构加大信贷投放、优化信贷结构，市内外金融机构新增贷款73.8亿元，其中市内金融机构新增贷款增速居全省第1位。适时转变帮扶企业的内容和方法，进一步提升服务企业的层次和水平，97%的规模以上工业企业恢复正常生产经营。积极采取多种措施鼓励和支持新生企业，新注册私营企业增幅创历史新高。企业生产经营的好转，使全市规模以上工业增加值增速逐月加快，从6月份开始保持两位数增长，全年增长15.3%。

　　(四)努力推动城乡统筹协调发展，城镇化进程进一步加快。大力推进新区南部片区基础设施建设和服务业发展，加快老城区和两县县城改造建设步伐，努力提升城市整体活力和实力，城镇化率预计达到49.6%。完成了城市总体规划修编、新区南部片区控制性详细规划等编制，构建新型城市骨架的方向和目标更加明晰。新区规划面积由38平方公里增加到65平方公里，涵盖金融、商务、会展、游乐等功能的产业综合功能服务区规划建设全面展开，淇水大道等一批城市基础设施项目竣工投用，市体育馆、艺术中心等一批社会公用事业项目和标志性建筑开工建设。7个城中村改造项目加快推进，村民居住环境和生活质量明显改善。职业教育、商贸物流、房地产等服务业加快发展，职业教育园区、综合物流园区等项目全面推进，产业综合功能服务区被确定为省服务业特色园区。老城区改造大力推进，"退二进三"工作有序展开，城市功能进一步提升，人民群众生活条件进一步改善。快速通道北延工程进展顺利，新老城区联系更加紧密。两县县城建设改造步伐加快，浚县工业化进程取得积极进展。鹤淇大道一期工程建成通车、二期工程启动，鹤淇产业带项目和园区建设、企业发展步伐加快。农业农村发展保持好形势，农业综合生产能力进一步提高，万亩高产创建核心示范区小麦、玉米平均亩产达到690.6公斤和858公斤，在全国率先实现万亩小麦、夏玉米一年两熟亩产吨半粮，为全省粮食核心区建设探索了经验、开拓了路子。修建农村公路373公里，改建农村公路桥梁20座，改善了农民的出行条件。新型农村社区建设取得新突破。

　　(五)强力推进产业集聚区规划建设，聚合优势和效应初步显现。统筹规划全市产业布局，科学明确功能定位，重点规划建设了宝山、鹤淇、金山、黎阳4个省级产业集聚区和山城区石林陶瓷、浚县粮食精深加工、鹤山区姬家山3个市级产业集聚区，在全省率先完成省级产业集聚区总体发展规划，并全部通过省评审。进一步拓宽投融资渠道，成立了市经济建设投资集团，组建了各县区融资平台，以加快基础设施建设、完善配套服务设施、促进项目落地为重点，全面加快产业集聚区发展。产业集聚区在建基础设施项目128个，完成投资50亿元；工业项目105个，完成投资112亿元，产业集聚区聚合力、吸引力进一步增强。鹤淇、宝山产业集聚区被确定为首批省对外开放重点产业集聚区，石林陶瓷产业集聚区被确定为省承接沿海地区陶瓷产业转移集聚区。现代化工、食品、汽车零部件、金属镁、消费电子等产业被列入省十大产业振兴规划，我市正在成为全省重要的现代化工、金属镁精深加工高新技术特色产业基地和食品产业集群。

　　(六)扎实推进循环经济和节能减排工作，生态文明建设成效显著。不断延伸循环经济产业链，积极构建循环型工业、农业和城市体系，加快推进总投资64亿元的33个循环经济项目，其中15个项目竣工投产并发挥效益，循环经济标准体系初步建立。大力推进既有建筑节能改造，被确定为国家可再生能源建筑应用示范市。加大节能减排和环境保护力度，万元工业增加值能耗降幅保持全省第一，各项环境保护省定目标提前圆满完成。实施"一河五园"保护利用开发工程，淇河森林公园、湿地公园、淇水诗苑、淇水乐园等开工建设。大幅度增加造林绿化投入，完成造林绿化26.9万亩，林木覆盖率、森林覆盖率达到25.1%和21.6%，林业生态市建设迈出新步伐。

　　(七)加快创新型鹤壁建设步伐，核心竞争力进一步增强。三年人才引进计划深入推进，人才智力支撑作用正在突显。加强了与中国科学院、上海交通大学等全国知名科研院所和高等院校在科技、人才、项目等方面的交流合作。大力实施职业教育攻坚计划，鹤壁职业技术学院新校区开工建设，对2009年初、高中毕业后未升学人员全部进行了职业技能培训。稳步实施科技创新计划，引进关键技术40项，12项国家、省级重大科技专项顺利实施，专利申请量增长34%。高新技术产业快速发展，高新技术企业增加值占工业增加值的比重由上年的11.6%提高到18%。成功创建省级可持续发展试验区。市农科院挂牌成立。高标准建成农业气象防灾减灾保障中心，被确定为全国现代农业气象防灾减灾体系建设试点。加快推进文化体制改革和文化产业发展，浚县被确定为全省文化改革发展试验区。

　　(八)认真抓好省"十大实事"和我市民生工程，人民群众生活条件继续改善。把改善民生作为经济发展的出发点和落脚点，解决了一批关系群众切身利益的实际问题。在全省率先启动并迅速推进惠及老城区19630户居民的地方棚户区改造工程，已拆迁棚户区39.4万平方米，开工建设住宅104.1万平方米，改造规模占总规模的27%以上；总建筑面积43.2万平方米的8643套廉租房正在加快建设，已竣工1070套，受到老城区人民尤其是直接受益群众的热情拥护和大力支持。城镇新增就业4.9万人，下岗失业人员再就业2.1万人，正在实现由劳务输出市向劳务输入市转变。各项社会保险工作均超额完成预定目标，城乡低保标准进一步提高，低保金按时足额发放。落实"两免一补"资金1.2亿元，惠及学生48.8万人次。新区湘江中学、福源小学、牟山小学建成。积极推进医药卫生体制改革，被确定为全省医药卫生体制改革试点市。新农合参合率达到99.4%，城镇居民基本医疗保险覆盖率达到90.9%。在全省率先为全市城乡居民建立了健康档案。积极开展手足口病、甲型H1N1流感防控工作，全市没有发生重大疫情。在全省率先创建"白内障无障碍市"，对5000名患者免费实施了手术。残疾人综合服务中心、特殊教育学校开工建设。市儿童救助中心建成投用。完成了22个乡镇文化站改扩建和673个文化信息资源共享工程基层服务网点扩展升级。人口自然增长率控制在4‰，稳定了低生育水平。解决了8.4万人饮水安全问题。涉及21个贫困村的30个扶贫开发整村推进项目全部完成。刁公岩、弓家庄移民搬迁工作基本完成。

　　(九)加强民主法制、精神文明和政府自身建设，发展环境进一步优化。坚持依法行政和依法治市，自觉接受人大及其常委会监督，支持政协参政议政，认真办理人大代表建议49件、政协提案214件，办结率100%。扎实推进平安鹤壁建设，有力维护了社会大局和谐稳定。政务公开工作深入推进。第三届社区居委会换届顺利完成。加强精神文明建设，公众道德水平和城乡文明程度进一步提升。认真组织开展深入学习实践科学发展观、营造风清气正好环境等活动，以提高执行力为抓手，进一步转变政府职能、改进工作作风，提高行政效能和公务员素质。下大力气开展中介机构与行政机关脱钩专项治理，将与行政机关挂靠的中介机构推向市场。创新并实行行政审批(许可)"两集中、三到位"，在全省率先实行项目联审联批工作机制，大幅缩短了审批时限、提高了行政效能。推进政府廉政建设，强化行政监察、财政、审计等监督，政府系统反腐倡廉工作取得新成效。国防和军队建设力度继续加大，双拥共建活动扎实推进，军政军民团结进一步巩固。外事、侨务、对台、民族、宗教、统计、档案、史志、人防、地震等工作取得新成绩。援助四川灾区重建工作进展顺利。

　　各位代表！

　　一年来，在极为困难的情况下，全市主要发展指标由全省靠后跻身全省前列，不少工作保持全省先进位次，多项重点工作在全省介绍了经验，既取得了显著的经济社会发展成果，又积累了在复杂环境中推动科学发展的宝贵经验。这是省委、省政府和市委坚强领导的结果，是市人大、市政协大力支持的结果，更是全市人民团结奋斗的结果。我们不能忘记全市上下争分夺秒、夜以继日迎战危机的顽强和艰辛，不能忘记广大干部职工脚踏实地、兢兢业业奋战在项目建设、招商引资、帮扶企业等工作一线的操劳和尽心，更不能忘记广大人民群众干好本职工作、支持全市发展的付出和坚韧。这种克难攻坚、实干苦干、顽强拼搏、无私奉献的精神，将激励我们在率先崛起的征程上阔步前进。在此，我代表市人民政府，向工作在全市各条战线的广大工人、农民、知识分子、干部、人民解放军指战员、武警官兵、公安民警以及各界人士致以崇高的敬意！向关心、支持我市现代化建设的同志们、朋友们表示衷心的感谢！

　　一年来，在战危机、保增长、保民生、保稳定的过程中，我们对全市经济社会发展存在问题的认识更加深刻：发展方式粗放、经济结构不合理的状况亟需改变，产业单、链条短、层次低、规模小的深层次矛盾尚未根本解决；老城区基础设施和公共服务设施欠账较多，低收入群体的生活水平亟待提高；浚县工业化进程尚未明显破题，改变农业大县、财政穷县面貌还面临很多困难；一些干部创新能力有待进一步提高，开放意识有待进一步增强，工作作风有待进一步改进。对此，我们一定高度重视，采取有效措施认真加以解决。

　　二、2010年政府工作总体要求和目标任务

　　今年是实施“十一五”规划的最后一年，也是夺取应对国际金融危机全面胜利、加快两个构建的关键一年。做好今年的工作，对于巩固经济回升基础、为"十二五"规划启动实施创造良好条件、推动我市跨越发展率先崛起意义重大。总体上看，今年我市发展面临着良好机遇。世界经济形势有望不断好转，国家继续实施积极的财政政策和适度宽松的货币政策，国际和沿海地区产业转移加快，中原崛起战略加快推进；经过长期以来特别是近年来的不懈努力，我市的产业发展有了一定基础，城市骨架持续拉大，一批重点项目先后落地并逐步发挥效益，全市人民干事创业的热情高涨，环境品牌逐步显现，这都为今后的发展创造了条件、打下了基础、备足了后劲。同时我们也应该看到，后危机时代不确定、不可预料因素增多，区域竞争更加激烈，保障和改善民生、发展社会事业、维护社会和谐稳定的任务依然艰巨。新的一年，我们将进一步统一思想、坚定信心、抢抓机遇，增强时代紧迫感和工作自觉性，更加努力扎实地推进各项工作。

　　今年政府工作的总体要求是：深入贯彻落实科学发展观，认真落实市委七届七次全会和市委经济工作会议精神，坚持既定发展思路和战略措施，继续以项目建设为主抓手，以转变发展方式和调整经济结构为主线，着力推进工业化、城镇化、农业现代化协调发展，加快增强经济实力、创新能力和综合竞争力，促进民生持续改善、社会和谐稳定，在加快两个构建、实现率先崛起进程中迈出新步伐。

　　主要目标是：生产总值增长11%；财政一般预算收入增长12%；规模以上工业增加值增长14%；全社会固定资产投资增长25%；社会消费品零售总额增长16%；外贸出口增长7%；引进市外直接投资100亿元；城镇居民人均可支配收入实际增长7%；农民人均纯收入实际增长6%；人口自然增长率控制在5.7‰以内；城镇新增就业3万人以上；单位生产总值能耗降低6%，化学需氧量、二氧化硫排放量完成省定目标；力争完成"十一五"规划目标；发展方式转变和经济结构调整取得重大进展。

　　主要任务是：

　　在产业培育壮大上迈出新步伐。着力抓好煤化工、食品、机械制造等战略支撑项目，加快推动金属镁精深加工、新型陶瓷、电子信息等产业发展，稳固壮大发展基础，加快提升产业层次。

　　在统筹城乡协调发展上迈出新步伐。全面推进新老城区规划建设管理，改善生态环境，打造城市文化，加快建设繁荣宜居的现代化城市。全面提升现代农业发展水平，深入推进社会主义新农村建设。

　　在创新型鹤壁建设上迈出新步伐。加大人才引进和培养力度，积极稳妥地推进改革创新，把推动发展的动力转移到科技创新和劳动力素质提高上来，努力为跨越发展、科学发展创造良好的体制机制环境。

　　在民生持续改善上迈出新步伐。以落实好省十项民生工程、办好我市民生工程为重点，切实做好就业再就业、社会保障、社会事业发展等事关人民群众切身利益的工作，不断提高人民群众的生活水平。

　　今年政府工作将认真贯彻落实省委、省政府"重在持续、重在提升、重在统筹、重在为民"的指导思想，切实做到不变调、不争论、不折腾、不懈怠，就是坚持加快两个构建、实现率先崛起的战略任务，把正在做的事情积极扎实向前推进；坚持按照既定的战略任务，同心同德、埋头苦干、狠抓落实；坚持聚精会神搞建设，一心一意谋发展，全心全意为人民；坚持以强烈的危机感和使命感，始终如一地奋力拼搏，保持已经形成的好势头、好局面。

　　三、2010年重点工作

　　围绕政府工作总体要求和目标任务，今年要继续坚持统筹城乡发展、统筹新老城区发展、统筹经济与生态、统筹发展与稳定，着力做好以下几项重点工作：

　　(一)进一步抓好项目建设，推进产业规模壮大和结构优化。着力抓好280个重点项目，力争完成投资233亿元，努力使今年成为项目建设全面提升的一年。突出抓好大项目。全力推进战略支撑项目，争取大型煤制烯烃项目尽早开工建设，电厂四期前期工作、大型预制构件项目取得实质性进展。强力推进24个煤电化材项目、11个陶瓷项目、22个食品加工项目、34个机械制造项目、12个镁冶炼及镁合金深加工项目、7个数码电子项目、3个光伏项目，加快打造全省重要的现代煤化工基地、陶瓷产业基地、食品产业集群、独具特色的汽车及汽车零部件基地、中国镁谷、电子产业集群等。认真做好南水北调中线工程、石武客运专线、山西中南部铁路通道、安新高速公路拓宽、鹤辉高速公路等项目的协调服务工作。突出抓好项目的竣工投产。整合优化各类资源，支持在建项目尽快建成并发挥效益。继续实施网络节点等管理方式，在确保工程质量的前提下，争分夺秒抓进度，千方百计赶工期，争取更多的项目竣工投产。突出抓好项目的结构优化。把项目建设与调结构、促转型更好地结合起来，在抓好工业项目、优化工业结构的同时，大力发展服务业。挖掘文化资源，延长文化产业链条，扶持推进一批文化产业重点项目和知名品牌，指导支持浚县文化改革发展试验区建设取得更大进展。加快推进旅游资源整合及战略合作，积极发展新型旅游业态，促进旅游产业加快发展。扶持现有商贸物流企业做大做强，大力引进知名商贸物流企业，继续抓好国际内陆港等项目，开工建设陶瓷综合市场、石林陶瓷物流园区等20个重点商贸物流项目。科学引导房地产市场，抓好一批重点项目，保持房地产业持续健康发展。突出抓好项目的推进保障。继续实行重点企业重点保护、重点项目重点督查，完善项目落地推进和协调服务机制，不断优化项目建设环境，确保项目顺利施工、早日竣工、早日见效。

　　(二)进一步加大招商引资力度，增强经济社会发展新动力。继续把招商引资工作摆在重要位置，坚持重点产业特殊政策、重点项目特殊服务、有所为有所不为，进一步提高招商工作的科学性、针对性和时效性，力争引进市外直接投资大幅增长，争取上级资金总额保持全省前列。大力引进行业龙头和产业集群。加强与国内外大企业的战略合作，积极承接沿海地区产业转移，突出抓好与中石化、中国电子、河南煤化集团、河南投资集团等知名企业的战略合作，务求在战略支撑项目引进上实现重大突破。注重商会协会招商、以商招商、集聚区招商、产业链和相关企业招商等，积极培育特色鲜明、竞争优势突出的产业集群。有效开展各类招商活动。有针对性地组织参加境内外招商推介活动，高标准谋划举办好我市的大型招商活动，争取再次举办金属镁、电子信息、新型陶瓷等全国性大型新产品、新技术展示推介会，使之常态化、多赢化。切实抓好招商引资项目谋划。继续安排项目专项资金，围绕国家、省产业政策和我市战略支撑、新兴先导产业，再谋划一批关联度高、成长性好、带动能力强的项目，力争新策划超亿元项目50个。及时与国家、省有关部门搞好对接，争取更多的项目、资金和政策支持。积极引导扩大民间投资。在教育、卫生、文化等领域推出一批项目，面向民间公开招标，在市场化、多元化融资上实现新突破。

　　(三)进一步加快产业集聚区建设，着力打造新的经济增长极。全面掀起产业集聚区建设发展高潮，力争全年完成投资100亿元以上，其中工业项目完成投资80亿元以上，新入驻超亿元工业项目25个。继续搞好产业集聚区规划。高标准推进产业集聚区总规和详规编制评审工作，一季度完成生产区、生活区、商务区等专项空间设计，上半年完成基础设施、公共服务设施、绿地及景观系统等专项规划。统筹抓好基础设施、服务设施和项目建设。加快集聚区道路、供排水、标准厂房等建设，完成起步区和近期发展区的基础设施建设。健全产业集聚区投融资平台、中小企业担保平台、土地开发整理中心，提升产业集聚区市场化运作能力。大力发展工业房地产、物流、人才培训等生产性服务业，支持建设科技研发、检测检验、公寓餐饮等公共服务平台，努力降低企业生产经营成本，提高产业集聚区承载力、吸引力和竞争力。结合产业集聚区定位，引导项目和企业围绕产业链加快集聚发展。进一步落实完善政策措施。认真落实对县域产业集聚区内符合国家产业政策的大工业用电项目执行省网直供电价政策，完善异地投资、税收分享政策，重点保障集聚区用地需求，环境容量指标优先配置入驻产业集聚区的项目。在完成区域环评的前提下，进一步简化入驻项目环评内容，发挥政策合力，促进产业集聚区又好又快发展。

　　(四)进一步提高服务企业水平，促进企业尽快做大做强。创新服务形式、丰富服务内容、提高服务实效，建立健全政策导向、市场开拓、融资服务、困难企业帮扶等长效机制。加快推进工业化和信息化融合步伐，引导、鼓励企业家进一步开阔眼界、开拓创新，促进企业战略重组、转型升级、加快发展。积极推进金融机构改革，优化金融生态环境，扩大中长期贷款、中小企业贷款规模和比例，使信贷增速高于生产总值增速。强化财政金融支持，采取财政补助、注入资产等措施，完善市级政府投融资体系，提高县区政府投融资平台融资能力，力争全市新增超亿元融资担保机构2-3家。规范担保机构运作，更好地发挥信用担保体系作用。大力培育和发展新企业，为经济发展不断注入新活力。

　　(五)进一步加快城镇化进程，推进城乡统筹协调发展。围绕加快构建新型城市骨架，坚持全域统筹谋划推进，加大体制机制改革和户籍等政策创新力度，促进新区、两县县城迅速膨胀人口，城镇化率提高到51%左右。继续提高城乡规划水平。高起点完善城市总体规划和新区南部片区、产业综合功能服务区等详细规划，编制完成老城区详细规划，编制城市供排水、供热、供气、交通、通信、环卫等公共设施推进计划。继续做好村镇体系规划编制工作。进一步提高规划服务水平，充分发挥规划的引导调控作用。高标准推进城市建设和管理。新区围绕强化产业、完善功能、提升品位，进一步提升建成区层次，以南部片区为龙头，加快续建项目建设，再开工建设一批基础设施项目，金融大厦等重要建筑及配套设施力争进入施工高潮，建成体现时代性、文化性、生态性的精品工程。加快推进7个城中村改造，再启动8个城中村改造，积极完善商业等服务设施。全面推进老城区改造，建设完善一批基础设施、公共服务等项目，高标准建设造型新颖、布局合理、适宜人居的新型社区，快速通道北延一期工程竣工通车、二期工程尽快开工建设，葛嘴线大修工程力争开工，进一步改善老城区环境、繁荣老城区经济。积极推进鹤淇大道二期工程，加快鹤淇产业带建设，推动鹤淇一体化发展。支持浚县县城进一步提高综合承载能力，加快吸纳聚集人口，尽早发展到20万人以上规模。以开通新乡-鹤壁城际公交为契机，加强与中原城市群的沟通协作。把以人为本与依法治理更好结合起来，全面加强城市管理尤其是老城区、两县县城的管理，探索完善长效机制，改进管理办法，提高管理实效，建设文明宜居的现代化城市。扎实推进新农村建设。增加并整合支农资金，统筹抓好农村特别是贫困乡村的道路、电网改造、饮水安全、通信、沼气入户等工程建设，解决农村居民饮水安全4万人；加大贫困村扶持力度，完成15个贫困村整村推进任务。结合工业化、城镇化发展，以产业集聚区农村整合和中心乡镇建设等试点工作为重点，强力推进新型农村社区建设。加快城乡社会事业发展。全面推行素质教育，促进义务教育均衡发展。认真实施校舍安全工程，确保师生安全。新区鹿鸣中学、福田中学、大赉店小学秋季招生，新开工建设2所中学、1所小学。大力实施食品药品放心工程，保障食品药品安全。加快推进医药卫生体制改革，扩大基本医疗保障制度覆盖面，初步建立国家基本药物制度，适时启动公立医院改革。健全基层医疗卫生服务体系，加快推进乡镇卫生院改造、社区卫生服务中心建设和标准化村卫生室完善提升。切实搞好重大传染病防控，提高应对突发公共卫生事件的能力。继续实施文化惠民工程，为24个乡镇文化站配备文化设施和设备，建设184个农家书屋，扶持建设30个示范性农村文化大院，办好第二届中国鹤壁民俗文化节，举办广场文化活动100场，加强文物保护管理。加快体育设施建设，市体育馆投入使用；广泛开展全民健身活动，培育竞技体育品牌。进一步抓好人口和计划生育工作，保持低生育水平，提高出生人口素质。

　　(六)进一步夯实现代农业基础，巩固和发展农业好形势。落实和完善强农惠农政策，加强粮食高产创建、基础设施建设、农业结构调整，注重运用先进科技、创新模式示范、搞好产业化经营，进一步提升现代农业发展水平。巩固扩大粮食高产创建成果。认真抓好万亩粮食高产创建示范区建设，进一步扩大规模，探索推广高产创建新模式，提升高产创建水平，打造我市现代粮食生产品牌，带动全市粮食增产增收。加强农业基础设施建设。着力抓好农田水利基本建设、农业综合开发、农业科技推广应用等，进一步改善农业基础条件，加强农资市场管理，提高保障能力。新增节水灌溉面积4万亩、高标准农田2万亩，改造中低产田2万亩。推进农产品质检项目和产业技术体系建设，提高农业标准化生产水平。加强气象防灾减灾、农业气象监测系统等建设，打造全国气象服务现代农业示范市。推进农业结构调整。大力推广规模环保养殖，新建或改造标准化蛋鸡、生猪养殖场30个、年出栏30万只标准化肉鸡养殖小区50个，大力发展畜产品精深加工，保持畜牧业在全省的领先水平，推进畜牧业大市向畜牧业强市转变。进一步发挥服务体系和专业合作社的作用，提高农民组织化程度，提升农业规模化经营水平。

　　(七)进一步加快创新型鹤壁建设，增强发展的动力和活力。强化支持创新的工作导向，加快由资源型城市向自主创新型城市转变，走创新驱动、内生增长之路。加大职业教育发展和人才引进力度。继续实施职业教育攻坚，建立政府引导、社会参与的多元化办学机制，加快职业教育园区、职业技术学院新校区等项目建设。扩大订单教育规模，打造特色专业，培育特色学校，树立职教品牌，提升办学层次和学生综合能力。加快实施三年人才引进计划，更好地发挥高层次人才的作用。努力提升自主创新能力。扎实推进可持续发展实验区建设。加快实施三年科技创新计划和粮食丰产科技工程，引进突破16项产业发展的核心、关键技术，组织开展4个重点科技攻关项目和重大科技专项。壮大自主创新主体，培育一批高新技术企业、创新型企业、科技创新团队和领军人才。丰富自主创新载体，培育壮大一批工程技术研究中心，把产业集聚区培育成创新型产业集聚区，把市农科院打造成集科研、生产、示范、推广于一体的农业科研创新龙头。积极实施质量立市战略。完善质量检验检测体系，健全质量监管长效机制，力争开工建设国家级金属镁检验中心。以实施名牌战略和标准化战略为重点全面提升产品质量，切实保障工程质量、改善环境质量、提升服务质量。

　　(八)进一步抓好生态文明建设，切实提高可持续发展能力。把生态文明放在更加重要的位置，努力实现发展与生态环保双赢。提升循环经济发展水平。健全并推广应用循环经济标准体系，进一步丰富内涵、提升层次。积极推进16个循环经济项目按计划开工，24个接续项目早日具备开工条件，抓好循环经济示范企业和示范园区建设，完善城市再生资源回收利用体系，开展低碳示范区试点工作，积极筹办循环经济论坛，把循环经济名片打造得更响更亮。坚持节约集约发展。加快推进31个可再生能源应用项目，抓好工业、建筑、交通、公共机构等领域节能工作，加大新型能源在建筑中的应用力度，继续对已有建筑实行节能改造，加快中水回用和节水工程建设，确保实现单位生产总值能耗下降年度目标和"十一五"总体目标，保持全省先进水平。进一步提高资源利用效率。坚持最严格的耕地保护制度和最严格的节约用地制度，科学规划城镇建设用地，鼓励建设多层标准化厂房，大力推进土地综合整治等，严控增量、盘活存量，严禁违法占地、粗放用地，确保重点项目建设用地，全面提高土地投资强度和产出效益。切实抓好环境保护。狠抓污染减排，严格实行主要污染物总量控制，集中环境容量保证市重点项目和重点产业发展。严把环保准入关，继续保持项目环评执行率和“三同时"率100%，确保过境断面和空气质量保持全省先进水平。继续加强淇河和水源地保护工作。加快污水垃圾处理和中水回用、污泥处置等设施建设。积极培育、大力扶持环保产业发展。深入开展绿色创建活动。大力推进生态建设。以”一河五园"建设为重点，更高标准、更大力度地推进林业生态示范市建设。抓好重点区域生态绿化，继续大力实施退耕还林，基本完成集体林地明晰产权工作，完成造林绿化19.6万亩，林木覆盖率提高到27%，让更多的土地披上绿色。

　　(九)进一步推进以改善民生为重点的社会建设，为人民群众谋取更多福祉。集中力量实施好省、市民生工程，多关注群众的所想所盼，多做雪中送炭的工作，下大力气解决好涉及人民群众切身利益的现实问题。加快保障性住房建设。坚持政府引导与市场化运作相结合的路子，更好更快地推进老城区棚户区改造，新开工安置住房82.7万平方米、竣工59万平方米。竣工廉租房7573套，新开工8438套，发放补贴1500万元以上。积极推进农村危房改造。全面扩大就业。继续实行积极的就业政策，发挥政府投资和重大项目带动就业的作用，提高中小企业、劳动密集型产业和服务业吸纳就业的能力。强化农村劳动力技能培训和初、高中毕业后未升学人员培训。建立就业供需定期沟通协调制度，提高公共就业服务的超前性、针对性和实效性。大力开展创建充分就业社区活动，进一步开发公益性岗位，妥善安排"4050"等困难群体就业，动态消除零就业家庭。大力宣传创业典型，加强创业辅导培训，鼓励支持劳动者自谋职业、自主创业，创建国家级创业型城市。继续提高社会保障水平。扩大各类社会保险覆盖面，解决好关闭破产企业退休人员和困难企业职工医疗保障等问题。做好新型农民合作医疗保险和城镇居民基本医疗保险工作，提高城乡医疗救助标准。积极开展新型农村社会养老保险试点。完善落实被征地农民补偿机制和社会保障制度，努力帮助解决就业等问题。加强城乡救助体系建设，提高救助水平和救助质量，对全市5.5万名低保对象分类施保，确保动态管理下的应保尽保。实施农村五保供养全覆盖工程。大力发展慈善事业。市残疾人综合服务中心竣工投用。争取成功创建全国无障碍城市。扎实推进精神文明建设。加快构建社会主义核心价值体系，大力弘扬以爱国主义为核心的民族精神和以改革创新为核心的时代精神。继续开展"讲文明、树新风"等活动，积极宣传表彰群众中涌现的先进典型和先进事迹。壮大志愿者队伍，丰富志愿者服务活动。深化文化科技卫生"三下乡"活动，提高城乡文明程度。全力做好社会稳定工作。深入推进社会管理创新，正确处理各类社会矛盾，健全调节利益关系的社会政策，形成依法有序表达诉求、及时有效解决问题的社会环境，保障群众合法权益。依法按政策妥善处理土地征用、拆迁安置、环境污染、劳资纠纷、涉法涉诉等方面的矛盾和问题。认真落实各项制度和措施，提高信访案件办理质量，扎实推进信访稳定工作。健全社会治安防控体系，依法防范和严厉打击各类违法犯罪活动。加强国防教育和国防后备力量建设，支持驻鹤解放军和武警部队建设，不断提高民兵预备役和人民防空建设质量，加快建设民兵综合训练基地；深入开展双拥共建活动，巩固发展军政军民团结。支持工会、共青团、妇联等人民团体参与社会管理和公共服务。同时，加强重大问题调查研究，科学编制"十二五"规划。

　　四、全面加强政府自身建设

　　开展好深入学习实践科学发展观和营造风清气正好环境集中教育活动，推进以“两转两提”为重点的服务型政府建设，切实做到全天候工作、全身心投入、全方位服务、全过程落实，提高领导和推进科学发展的能力，打造更具竞争力的环境品牌，营造更加浓厚的发展氛围。

　　深入推进依法行政。牢固树立法治观念，积极开展依法治市工作暨法治鹤壁创建活动，依照法定权限和程序管理经济社会事务。严格执行人大及其常委会的决议、决定，自觉接受人大法律监督和政协民主监督，认真办理人大代表建议和政协提案，充分听取各民主党派、工商联、无党派人士及各人民团体的意见，广泛接受社会公众和新闻舆论监督。依法推进政务公开，更加注重公权力大、公益性强、公众关注程度高的部门和领域的信息公开。规范执法行为，完善行政问责制度，坚决防止和克服行政慢作为、不作为和乱作为。

　　继续转变政府职能。积极稳妥推进政府机构改革，更好地履行经济调节、市场监管、社会管理和公共服务职能。推进事业单位分类改革、事业单位岗位管理制度改革等。进一步减少和规范行政审批事项，健全联审联批等便民措施。完善覆盖市县两级的行政效能电子监察系统，实现对行政审批、行政征收、行政处罚、公共资源交易等施政情况的实时监控、预警纠错和绩效考评，不断提高政府部门及工作人员的服务意识和水平。

　　着力提高行政效能。进一步强化学习，注重学习业务知识，提高做好本职工作、应对突发事件、运用市场手段等方面的能力；注重学以致用，采取培训考察等有针对性的形式和方法，提高执行能力；注重营造氛围，努力打造学习型机关和学习型干部队伍。进一步强化责任，教育引导政府系统广大干部职工增强责任感、使命感，尽心尽力、创造性地履行好工作职责，切实做到责随职走、心随责走，高质量高标准地推进工作。进一步强化考核督查，健全激励约束机制，对重点工作明确责任单位、工作步骤、时间节点和具体要求，确保政令畅通、令行禁止，确保各项工作取得实效。

　　进一步改进工作作风。更加注重民主决策，充分利用政府网站、12345市长热线等渠道了解民情民意，确保各项决策符合实际、符合人民群众意愿。更加注重实效，做到出实招、下实劲、办实事，多干少说、说到做到、说好办好；投入更多的时间和精力深入群众、深入发展一线，加强调查研究和指导督查；凝神聚力、不图形式，减少应酬、排除干扰，一以贯之、狠抓落实，务求经济社会发展取得实效、人民群众得到实惠。更加注重廉政建设，大力弘扬艰苦奋斗精神，努力做到勤俭节约办事，坚决制止铺张浪费现象；完善惩治和预防腐败体系，严肃查处违纪违法案件，努力让每一项权力在阳光下运行，维护政府系统干事创业、公正廉洁的良好形象。

　　各位代表！

　　面对发展改革的新形势，面对全市人民的新期望，我们深感责任重大、使命光荣。让我们在省委、省政府和市委的正确领导下，深入贯彻落实科学发展观，坚定信心、抢抓机遇、团结奋进、拼搏实干，谱写加快两个构建、实现率先崛起的新篇章！
